# Effect of teaching with or without mirror on balance in young female ballet students

**DOI:** 10.1186/1756-0500-7-426

**Published:** 2014-07-04

**Authors:** Angela Notarnicola, Giuseppe Maccagnano, Vito Pesce, Silvia Di Pierro, Silvio Tafuri, Biagio Moretti

**Affiliations:** 1 Course of Motor and Sports Sciences, Department of Neuroscience and Organs of Sense, Faculty of Medicine and Surgery, University of Study of Bari, Lungomare Starita 1, 70123 Bari, Italy; 2 Orthopedics Section, Department of Neuroscience and Organs of Sense, Faculty of Medicine and Surgery, University of Study of Bari, General Hospital, Piazza Giulio Cesare 11, 70124 Bari, Italy; 3 Department of Biomedical Sciences and Human Oncology, Faculty of Medicine and Surgery, University of Study of Bari, General Hospital, Piazza Giulio Cesare 11, 70124 Bari, Italy

**Keywords:** Dancing, Postural balance, Mirror neurons, Feedback, Sensory

## Abstract

**Background:**

In literature there is a general consensus that the use of the mirror improves proprioception. During rehabilitation the mirror is an important instrument to improve stability. In some sports, such as dancing, mirrors are widely used during training. The purpose of this study is to evaluate the effectiveness of the use of a mirror on balance in young dancers. Sixty-four young dancers (ranging from 9–10 years) were included in this study. Thirty-two attending lessons with a mirror (mirror- group) were compared to 32 young dancers that attended the same lessons without a mirror (non-mirror group). Balance was evaluated by BESS (Balance Error Scoring System), which consists of three stances (double limb, single limb, and tandem) on two surfaces (firm and foam). The errors were assessed at each stance and summed to create the two subtotal scores (firm and foam surface) and the final total score (BESS). The BESS was performed at recruitment (T0) and after 6 months of dance lessons (T1).

**Results:**

The repeated measures ANOVA analysis showed that for the BESS total score there is a difference due to the time (F = 3.86; p < 0.05). No other differences due to the group or to the time of measurement were found (p > 0.05). The analysis of the multiple regression model showed the influence of the values at T0 for every BESS items and the dominance of limb for stability on an unstable surface standing on one or two legs.

**Conclusions:**

These preliminary results suggest that the use of a mirror in a ballet classroom does not improve balance acquisition of the dancer. On the other hand, improvement found after 6 months confirms that at the age of the dancers studied motor skills and balance can easily be trained and improved.

## Background

Postural stability is crucial in maintaining body balance during quiet standing, locomotion, and any activities that require a high degree of balance performance. Motor activity based on motor-skill learning, particularly dance, can benefit balance [1]. Dance involves everything from listening to music to find the beat, looking at oneself in the mirror to memorize choreographed fight, apart from its requirements for physical activity.

Previous studies have found that dancers are better at learning new motor sequences when they watched movements that were visually familiar, but have never executed [2,3]. The researchers suppose that this ‘mirror system’ integrates observed actions of others with an individual’s personal motor repertoire, and suggest that the human brain understands actions by motor simulation [4]. In dance training and performance, mental imagery of movement is frequently used as a tool for learning and optimizing movements [5]. Dancers use mental imagery in creating new material [6], to exercise the memorization of long complex phrases, and to improve movement quality in terms of spatio-temporal adaptation and artistic expression. Dance training has been found to increase the amount and efficiency of kinesthetic imagery used and to enhance the imagery of kinesthetic sensations, making images more complex and vivid [7].

Dancing allows for a good commando of static and dynamic balance. Static balance is important when properly executing specific positions. For the “en pointe” position, where dancers balance on their pointed toes dancers need to maintain a vertical position with a reduced surface [8]. When dancers prepare for “a port de bras” while standing center floor in first position, the anticipated movement of the arms disturbs the static standing body [9]. The brain senses this intent to move and activates the muscles of the trunk and legs shortly before the onset of arm movement to prevent falling. Similarly, when preparing to “tendu”, the reflex muscle synergies in the trunk and standing leg activate to maintain balance milliseconds before the gesture leg moves forward. However, hand dynamic balance also plays an important part in dance particularly in the execution of spins (“pirouette”) [10]. It is considered a complex task, involving a strategy of head movement, the marking of the head, which dissociates the rotation of trunk and head, while the body spins, the eyes stare at an established point, and when the maximal cervical rotation is reached, the head performs a fast rotation towards the same direction of the movement, and then the eyes stare at the same point again. Literature reports that dancers have higher balance in comparison to athletes of other sporting activities and non-sporting subjects [11-14]. This can be justified by better control of the position of the upper limbs and less postural oscillation. Virtually any dance style challenges balance: one legged stance with eyes moving or closed at the ballet barre; leaping with quick directional or level changes with arms opposing legs in modern; and falling or rolling in contact improvisation or other dance forms. Probably these motor strategies are determined by the specificity of the training for the dance, like the use of mirrors and continuous visual control [10]. Pailhours showed that young student dancers are more dependent on vision than older students [15]. The difference in the equilibrium reactions of these two groups may be due more to a higher level of skill than a difference of maturation of the balance sensorimotor system. Golomer et al. [16] found that the contribution of vision in balance of dancer differs according to age: for 14-year-old students the postural control was less visually dependent than for 11-year-old student dancers, while 18-year-old dancers, although professional, were more dependent on vision than 14-year-old student dancers. A possible explanation of the dependence on vision of the 18-year-old dancers is the perturbation of the trunk proprioceptive reference linked to growth acceleration reduces information for postural control with respect to vestibular and visual references.

In recent years some authors have developed hypotheses about the negative effects of mirror use during dance lessons. Radell et al. [17] suggest that the use of the mirror in a ballet classroom may negatively affect the skill acquisition of a dancer. Some years later, the same authors concluded that while the use of a mirror has some benefits in training, higher performing dancers feel better about their body image when they do not use a mirror [18]. During rehabilitation a mirror is used as an instrument to improve stability [19]. For this reason we carried out this study to see if mirror use permits better stability and movements during dance lessons.

## Methods

We set up a clinical randomized prospective study designed to recruit volunteer younger female dancers, halfway through the lesson season. Subjects recruited were all students who attended classical dance lessons for 1 hour a day, 2 days a week. All parents read and signed the informed consent form approved by the local Ethics Committee of Bari University General Hospital, which also approved the study procedures. Subjects who had suffered a musculoskeletal injury to a lower extremity or a head injury were excluded from the study. We screened subjects for any pre-existing visual, vestibular, or balance disorders through self-report. Any subjects were randomly assigned to one of the two test groups, mirror-lessons or non-mirror-lessons groups. The teacher was always the same. During 6 months the students attended the same lessons (1 hour a day, 2 days a week) that were different only because they were in front of mirror (mirror-lessons group) or turned away from mirror (non-mirror-lessons group). The posture stability was valued at the recruitment (T0) and after 6 months (T1). According to the inter-rater and intra-rater reliability for the total BESS scores of 0.57 and 0.74 respectively [20], each subject took the test twice and two independent investigators measured the error score separately. The BESS score for each subject was expressed as an average.

### Balance error scoring system

**Figure 1:**
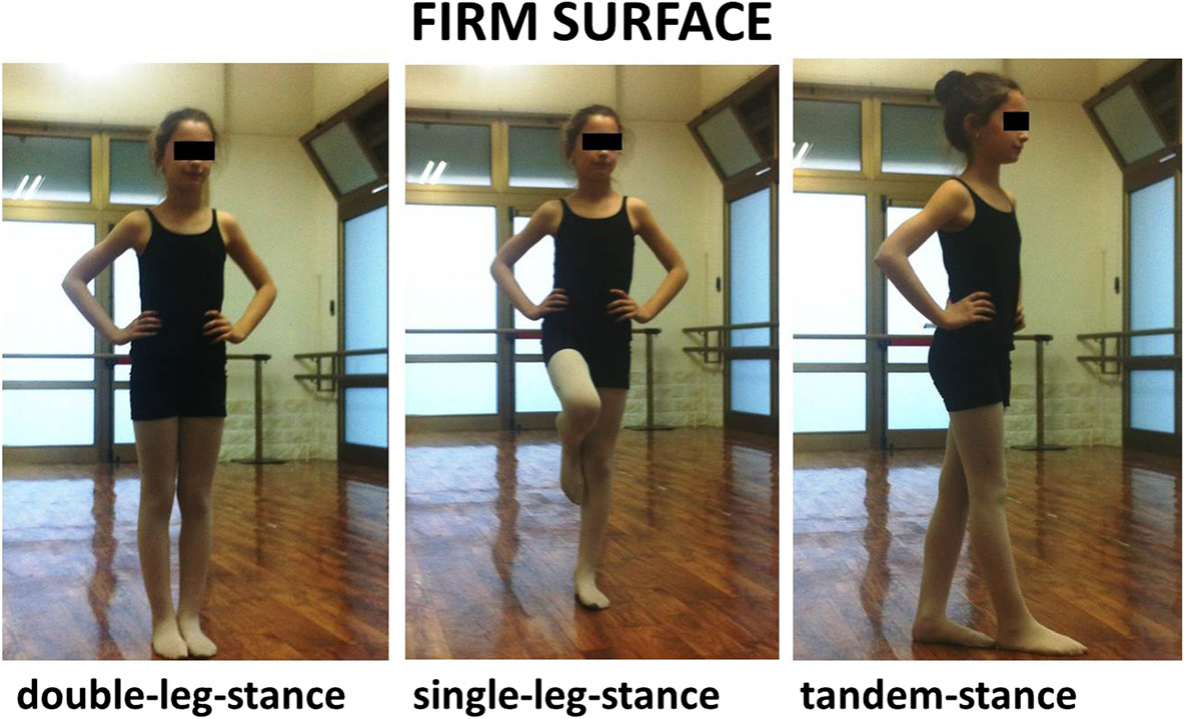
**The three positions on FIRM SURFACE of BESS score.** We received consent from the parent to publish the photo of the dancer.

**Figure 2:**
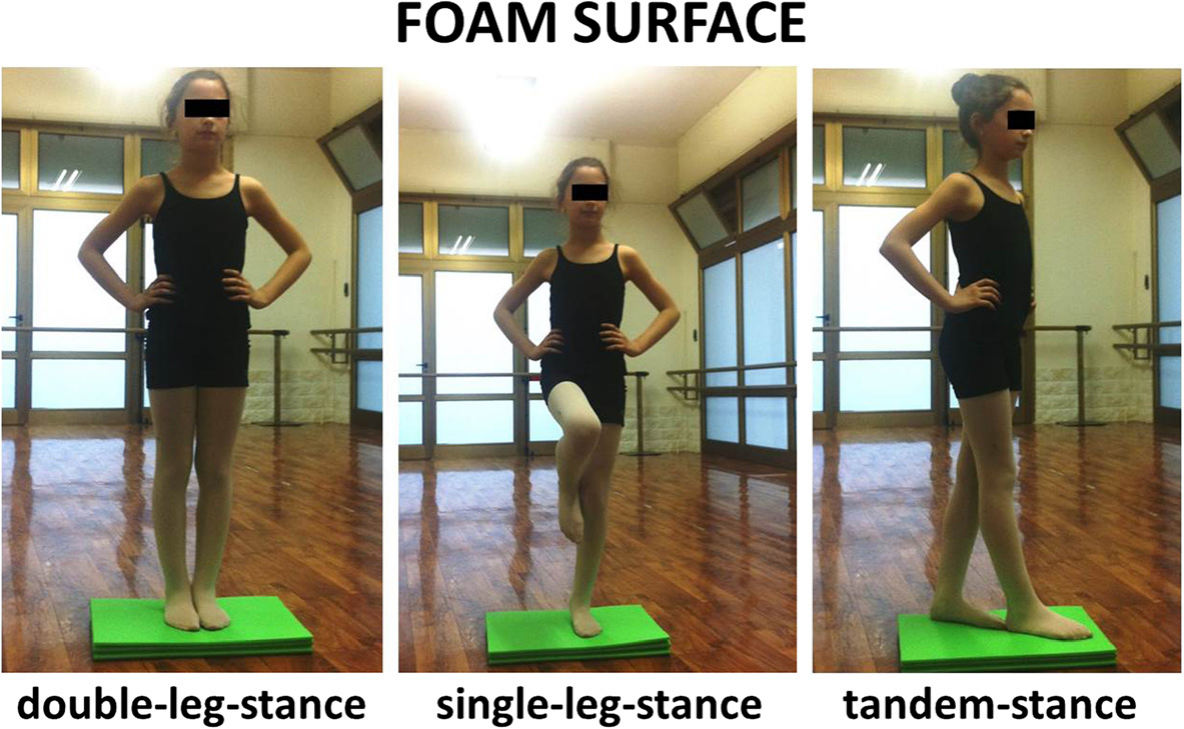
**The three positions on FOAM SURFACE of BESS score.** We received consent from the parent to publish the photo of the dancer.

Postural stability was measured using BESS (Balance Error Scoring System) error scores. The BESS comprises 6 conditions: double-leg, single-leg, and tandem stances on firm and foam surfaces (Figures 1 and 2). The firm surface was the floor of a ballet academy. The foam surface consisted of a 46 × 46 × 13-cm block of medium-density foam on a 10-cm-thick. A stopwatch was used to time each of the 20-second trials. One BESS error was scored if the subject engaged in any of the following: (1) lifting the hands off the iliac crests; (2) opening the eyes; (3) stepping, stumbling, or falling; (4) moving the hip into more than 30° of flexion or abduction; (5) lifting the forefoot or heel; or (6) remaining out of the test position for longer than 5 seconds. Error scores were calculated for each of the 6 conditions and summed to obtain the total BESS score. A full description of BESS scoring and reliability has been previously published [21-27]. Before the test, subjects were allowed to familiarize themselves with the different conditions. They were first allowed to try standing on the firm surface. Once they were comfortable standing on each surface, we then instructed them in the correct positioning for each of the 6 conditions. The double-leg stance conditions consisted of the subject standing with feet together. The single-leg stance was performed on the non-dominant leg, as determined by which limb the subject would not preferentially use to kick a ball. The dominant leg was positioned so that the hip was flexed to approximately 30° and the knee flexed to 90°, leaving the foot approximately 25 cm off the ground. We instructed the subject not to lean the dominant leg on the non-dominant leg. The non-dominant foot was positioned behind the dominant foot in the tandem stance, and the subject was instructed to maintain the stance with the big toe of the non-dominant foot touching the heel of the dominant foot. For all conditions, we instructed the subject to remain still with their eyes closed and hands on their hips. After the instruction, each subject was given 2 familiarization trials on each condition before the actual data collection. During the familiarization and testing sessions, each condition lasted 20 seconds, and at no point was the clock stopped. We instructed the subject to remain as still as possible; if she moved from the test position, she was to return to it as soon as possible. During the testing, the examiner was positioned 3 m away from the subject, so the subject’s eyes, hands, and feet could all be observed.

### Power analysis and statistical analysis

Given the previous data in literature related to mean and standard deviation of the number of BESS errors at recruitment and follows up [21-27], we established alfa = 0.05 and power = 0.90 and yielded a minimum number of 24 subjects per group. For each subject a file was completed with demographic variables (age, weight, height, dominant limb, how many years attends). Leg dominance was determined by methods used in previous studies [28,29]. The information was put into a database with FileMaker pro software which was analyzed using STATA MP11 software. We used averages and standard deviation to quantify the group. To compare the means of the two groups (mirror and non-mirror) in the two times (T0 and T1) we used a model of ANOVA repeated measures. Qualitative variables were expressed as proportion and for the comparison of the proportion we used chi-square test. To evaluate the association between the variables measured at T1 and T0 values, age, weight, height, dominant limb, time of practiced group we built a multiple logistic regression model. For each test we considered p < 0.05 to be significant. In the models of multivariable analyses as a reference point we used the T1 values and as determine/confounding factors a T0 values, age, weight, height, dominant limb, time of activity and group.

## Results

**Figure 3:**
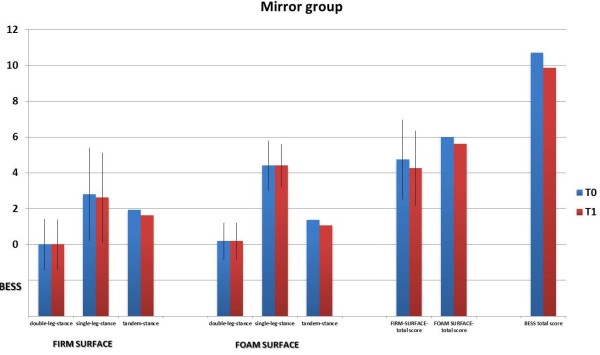
The graph of mean values of BESS errors at recruitment (T0) and after 6 months (T1) in the mirror lesson group.

**Figure 4:**
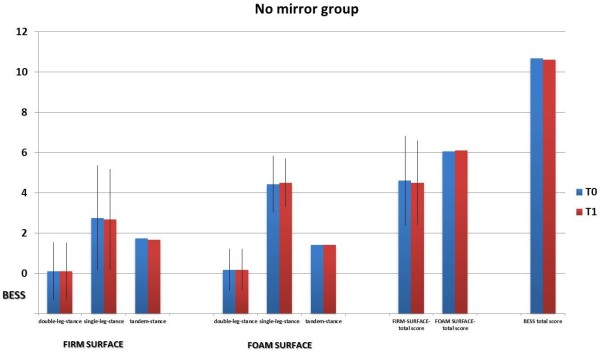
The graph of mean values of BESS errors at recruitment (T0) and after 6 months (T1) in the no mirror lesson group.

There were 64 subjects in our study sample, 32 in the mirror group and 32 in the non-mirror group; the average age is 9.6 ± 0.5 years (range 9–10 years), without differences between the two groups (t = 1.1; p = 0.15). The average weight was 32 ± 5.4 kg without differences between the two groups (t = 1.1; p = 0.14) and the average years of activity in both groups was 4.6 ± 1.4 (t = 1.4, p = 0.09). 10% of this subjects recruited had right main limb and these proportions were similar in two groups (chi-square test = 0.37; p = 0.54).Figures 3 and 4 show the mean values and the standard deviations of subtotal scores and total score of the BESS-total score in the two groups at T0 and T1. The repeated measures ANOVA analysis shows that for BESS total score there is a difference due to the time (F = 3.86; p < 0.05). No other differences due to the group or to the time of measurement were found (p > 0.05).

The analysis with the logistic regression model highlights that the values at T1 of Firm Surface-single-leg-stance (coef = 0.96; t = 12.3; p < 0.0001), of Firm Surface-tandem-stance (coef = 0.89; t = 13.3; p < 0.0001), of Firm-Surface-total score (coef 0.85; t = 13.3; p < 0.0001), of Foam Surface-tandem-stance (coef = 0.91; t = 8.9; p < 0.0001), of Foam Surface-total score (coef = 0.99; t = 12.7; p < 0.0001) and of BESS-total score (coef = 0.81; t = 11.6; p < 0.0001) are influenced by the corresponding values at T0.

The values of Foam Surface-double-leg-stance and of Foam Surface-single-leg-stance at T1 are influenced by the same value at T0 (respectively coef = 0.80, t = 6.59, p < 0.0001; coef = 0.98, t = 13.3; p < 0.0001) and by right dominance (respectively coef = 0.45, t = 3.12, p = 0.005; coef −0.93, t = 3.67, p = 0.001).

## Discussion

In our work we studied the effects of balance on visual feedback linked to mirror use or non-mirror use during ballet lessons. Balance is a complex function achieved by multi-sensory integration of visual, vestibular, and somesthetic afferences, central motor control, and context-specific response generation [30]. In detail, the stimulation of visual analysis allows us to build kinesthetic memory [31]. An example of this memory is when we reach out, without looking, for an object that we have placed nearby. We have a motor plan based on previous memorized visual information. It is not known yet whether the neural mechanisms for reaching a position defined by kinesthetic cues share a common spatial frame of reference with the mechanisms for reaching memorized visual targets. Starting from the earliest literature on motor memory [32,33], recent studies are interesting to verify the “mirror” neurons [34-36]. Within the pre-motor and parietal cortices of the macaque monkey, “mirror” neurons have been recorded which discharge both when the monkey performs an action, and also when observing the experimenter or another monkey performing the same action [34-36]. A similar mirror system may exist in corresponding areas of the human brain [37-39]. Some reward-related areas in the brain are connected with motor areas and mounting evidence suggests that we are sensitive and attuned to the movements of others’ bodies, because similar brain regions are activated when certain movements are both made and observed. Using functional magnetic resonance imaging (MRI) it was found the motor regions of professional dancers’ brains are more active when they watch other dancers compared with people who don’t dance [40]. The researchers suppose the network of motor areas involved in preparation and execution of action are also activated by the observation of actions.

In recent years mirror feedback has been studied in the rehabilitation field. Mirror therapy is applied to different neurologic and orthopedic diseases. In elderly adults instability decreased, in particular in medial-lateral direction [41]. In elderly trans-femoral amputees the upright stance control improved [42]. In patients with chronic stroke, MRI results showed a shift in activation balance within the primary motor cortex toward the affected hemisphere [43].

In our study, based on what happen in rehabilitation [41-43], we hypothesized that the use of mirror could allow the young dancers to improve their balance during the sensitive phase of the acquisition of motor skill. We administered the Balance Error Scoring System (BESS) which evaluates the mistakes in maintaining stability with closed eyes in 3 different positions and each position must be maintained on the stable surface and then an unstable surface.

The BESS is a standardized, rapid, inexpensive screening test of postural stability that can be helpful for documenting stability [26]. It has been used in many studies with healthy athletes, and as an outcome measure relating to low limb instability or those completing neuromuscular training [21-27,44,45]. At the beginning this test was used to study balance stability [46]. The average number of BESS errors depends on the stance and surface [45]. Very few errors are associated with the double-limb stance on either the firm or foam surfaces [24]. The single-leg stance is the most responsible for adding errors to the total BESS score on the firm and on the foam surface [24]. Less errors are added to the total BESS score during the tandem stance on the two surfaces [24].

Ambegaonkar and co-workers studied the balance in dancers using different scores [46]. The Balance Error Scoring System (BESS) and the Star Excursion Balance Test (SEBT) showed a better balance in the dancer than the non-dancer [46]. When authors compared the dancers’ scores to balance in athletes, the dancer participants’ BESS scores were not better than those of soccer, baseball, basketball, or gymnastics athletes [47]. This observation of similar scores between athletes and dancers was surprising, because it is suggested that dancers have better balance, due to their training [48]. In a recent review the researchers found that BESS is sensitive to verify the improvement after training [45]. On the other hand, up to now no study has verified that after a period of specific training dancers undergo an improvement of BESS. However, the use of similar scores have shown that in dancers significant changes of balance may occur in relation to different training conditions [49].

In our study both groups were matched for epidemiological characteristics and BESS values at the recruitment. The errors of stability linked to position and surface were the same that we found in literature [21-27]. At T1 the BESS score improved for both groups, without any between-group difference. This improvement is due to the time. These results did not support the initial hypothesis that mirror training would be more effective to improve balance compared non-mirror training, consistent with a recent clinical study in which the use of a mirror was proved not to be useful in improving balance [50]. The lack of between-group difference could probably be due to the short duration of administration of mirror visual feedback. Indeed, in our study the number of weekly hours with mirror training was lower compared to previous studies [50-56].

In our study, the improvement in balance was due to the time. This result is consistent with sensitive development phases, that is, times when any skill, if worked on, can be better improved compared to other periods of time [57]. The age of the subjects in our study were in that sensitive age range for balance, which is between 9 to 12 years.

The analysis of the multiple regression model showed how right dominance could be a confounding factor and at the same time to increase the number of errors during the test on an unstable surface with double or single bearing. This can be explained by the notion that humans are generally right-footed for mobilization tasks, but left-footed for tasks requiring postural stabilization [58,59].

The main limitation of the study is the fact that dancers train for static and dynamic balance [60], but we tested only for static balance. In future studies, the Star Excursion Balance Test, to measure dynamic balance, and a stabilometry, to quantify the dislocation of the center of pressure, could be used. Until now, in dance static balance has been studied with the stabilometry. The BESS has been used mainly for other sports athletes or for subjects with lower limb instability. Therefore, we do not have the reference values of the BESS in a larger population of dancers.

### Clinical implications

Our preliminary data shows that mirrors were not effective in improving balance. The findings of this study could have important implications for dancers, teachers, and medical staff, allowing them to exclude the use of a mirror while planning their work programme. However, we should still consider that in previous works there have been results found in favour of mirror use [19,41-43,50-55]. We believe that applying this intervention for a longer period of time (perhaps continuing visual feedback at home after a lesson), could be beneficial in improving the effects and outcome. Furthermore, the age of the subjects could also influence results. If on one hand we consider that the subjects examined were in that phase in which balance is more easily trained, but on the other hand we should also consider the immaturity of the subjects’ visual feedback which finishes developing after 18 years of age. We can assume that the maturity of the nervous system justifies the benefits found in previous studies related to mirror therapy in adulthood [19,41-43,50-56].

## Conclusions

We verified that female dance students did not improve their static balance when they had lessons with the use of the mirror. Future studies are necessary in order to clarify whether the use of a mirror could be beneficial in both teaching and medicine.

### Availability of supporting data

The data is deposited at University of Bari, Course of Motor and Sports Sciences (Silvia Di Pierro’s thesis of degree).

## Competing interests

The authors declare that they have no competing interests.

## Authors’ contributions

AN, GM and BM drafted of the manuscript and reviewed the literature. AN, VP and SDP conceived the study, participated in its coordination and in the acquisition of the data. ST was involved in analysis and interpretation of the results and in statistical analysis of the data. All authors read and approved the final manuscript.
